# Sonochemical Preparation of a Magnet-Responsive Fe_3_O_4_@ZIF-8 Adsorbent for Efficient Cu^2+^ Removal

**DOI:** 10.3390/nano12050753

**Published:** 2022-02-23

**Authors:** Trung Tuyen Bui, Duc Cuong Nguyen, Si Hiep Hua, Hyungphil Chun, Yong Shin Kim

**Affiliations:** 1 Institute of Natural Science and Technology, Hanyang University, Ansan 15588, Korea; trungtuyen@hanyang.ac.kr; 2 Department of Bionano Engineering, Hanyang University, Ansan 15588, Korea; cuongnguyen@hanyang.ac.kr; 3 Graduate School of Applied Chemistry, Hanyang University, Ansan 15588, Korea; huasihiep@hanyang.ac.kr; 4 Department of Chemical and Molecular Engineering, Hanyang University, Ansan 15588, Korea

**Keywords:** sonochemistry, Fe_3_O_4_ nanoparticle-embedded ZIF-8, magnetic composite, copper adsorption

## Abstract

This work presents a novel approach to synthesizing magnetic core-shell nanocomposites, consisting of magnetic nanoparticles and a metal-organic framework, for environmental applications. The synthesis is based on the encapsulation of magnetic Fe_3_O_4_ nanoparticles with microporous zeolitic imidazolate framework-8 (ZIF-8) nanocrystals via ultrasonic activation under a continuous supply of precursor solutions. This sonochemical approach is proven to be a fast, cost-effective, and controllable route for the preparation of magnet-responsive Fe_3_O_4_@ZIF-8 nanoparticles with a core-shell structure. The functional nanomaterial possesses a high content of ZIF-8 and combined micro/mesoporosity, and thus can be used as adsorbents that can be easily separated using a magnet. In particular, the sonochemically prepared Fe_3_O_4_@ZIF-8 exhibits significant adsorption performance for the removal of copper ions from water: a short adsorption time (10 min), high maximum uptake capacity (345 mg g^−1^), and excellent removal efficiency (95.3%). These performances are interpreted and discussed based on the materials characteristics of Fe_3_O_4_@ZIF-8 established by microscopy, gas sorption, X-ray diffraction, and thermal analysis.

## 1. Introduction

The contamination of natural freshwater by heavy-metal species is one of the most serious and persistent environmental problems. Toxic heavy-metal pollutants have an adverse effect on the underwater ecosystem and cause damage to cellular organelles, which are often related to various diseases, birth defects, or even mutations [1,2]. For example, copper (II) ions are known to be toxic and carcinogenic when ingested in large amounts, causing cramps, diarrhea, liver failure, kidney damage, and abdominal pain [3,4]. To date, various methods were suggested and are being used to remove hazardous heavy-metal species from aqueous solutions, including chemical precipitation, ion exchange, flotation, ultrafiltration, electrodialysis, and adsorption [2,5,6,7,8,9]. Among them, adsorption is regarded as one of the most effective and attractive methods. It has benefits associated with high uptake capacity, absence of chemical sludge, and low cost of operation owing to the flexibility and simplicity of the system design. Conventional heavy-metal adsorbents are activated carbon [10], zeolites [11], and chitosan [12]. However, these adsorbents typically suffer from low adsorption capacities and removal efficiencies. Recently, carbon nanomaterials, such as carbon nanotubes [13] and graphene oxide [14], were investigated as promising heavy-metal adsorbents with improved adsorption characteristics. However, the relatively high production cost and toxicity of these carbon nanomaterials restrict their practical use as adsorbents in water remediation and wastewater treatment.

Zeolitic imidazolate framework-8 (ZIF-8) is a water-stable metal–organic framework (MOF) with well-defined micropores and framework structure. ZIF-8 nanoparticles were extensively studied and proven to be an emerging adsorbent for the removal of heavy-metal ions from aqueous solutions owing to their stabilities over wide pH ranges, feasible chemical interactions at the Zn and N sites, and high specific surface area [15,16]. However, the separation of small ZIF-8 nanoparticles after the adsorption process is a time-consuming and labor-intensive process, hampering their usage as an adsorbent. Thus, magnetic ZIF-8 composites attracted considerable attention for the facile separation of ZIF-8 nanocrystals from solutions. A typical example is a magnetic Fe_3_O_4_ nanoparticle-embedded ZIF-8 composite with a core-shell structure (Fe_3_O_4_@ZIF-8). Several synthetic routes were proposed for the preparation of Fe_3_O_4_@ZIF-8 composites. Most of them are based on a one-pot solvothermal synthesis of ZIF-8 in the presence of pre-prepared Fe_3_O_4_ nanoparticles [17,18,19,20,21]. This simple strategy, however, results in a limited loading of the microporous component in Fe_3_O_4_@ZIF-8. Recently, Zheng et al. [22] subsequently performed the solvothermal synthesis several times to achieve a higher ZIF-8 loading because the adsorption capacity strongly depends on the relative amount of active ZIF-8 in the composite. The repetitive synthesis requires a long process time and large amounts of chemicals. Therefore, it is still a challenge to develop a rapid and cost-effective synthesis method for magnetic Fe_3_O_4_@ZIF-8 adsorbents with a high ZIF-8 content because high-quality Fe_3_O_4_@ZIF-8 nanoparticles are also very promising in other fields such as drug delivery [19], bioimaging [20], and heterogeneous catalysis [21].

The use of ultrasound is very useful for enhancing the reaction rate in a variety of chemical reactions owing to the localized high temperature and pressure caused by cavitation of the liquid medium [23]. Therefore, the sonochemical synthesis could be an efficient method for obtaining not only fine chemicals, but also complicated nanomaterials under mild conditions. The ultrasound-assisted method was proven to be a facile, environmentally friendly, and versatile synthetic approach for the preparation of microporous MOFs [24]. Indeed, the sonochemical synthesis of high-quality ZIF-8 nanocrystals was previously reported on a large scale [25] or at low temperature [26]. However, the sonochemical preparation of hybrid ZIF-8 nanomaterials with controlled size and well-defined microstructures remains a significant challenge.

Herein, we report a novel strategy of using the sonochemical growth of ZIF-8 crystals on Fe_3_O_4_ nanoparticles to attain magnet-responsive Fe_3_O_4_@ZIF-8 adsorbent with a higher ZIF-8 content. When compared with that of the Fe_3_O_4_@ZIF-8 composite (*c*-Fe_3_O_4_@ZIF-8) synthesized using a conventional solvothermal method, our sonochemical Fe_3_O_4_@ZIF-8 nanoparticles exhibit a higher ZIF-8 content of 66 wt.% under the same reaction time of 30 min. The product possesses a high surface area of 896 m^2^ g^−1^ and superparamagnetism. Furthermore, our Fe_3_O_4_@ZIF-8 adsorbent exhibits excellent performance for the removal of harmful Cu^2+^ ions from water with the specific uptake of 345 mg g^−1^ and 97% saturation within 10 min.

## 2. Materials and Methods

### 2.1. Chemicals

Zinc nitrate hexahydrate (Zn(NO_3_)_2_·6H_2_O), 2-methylimidazole (Hmin), ethylene glycol, polystyrene sulfonate (PSS) sodium salt, and copper nitrate trihydrate (Cu(NO_3_)_2_·3H_2_O) were purchased from Sigma–Aldrich. Iron(III) chloride hexahydrate (FeCl_3_·6H_2_O) and sodium acetate (NaOAc) were acquired from Junsei Chemical (Tokyo, Japan) and Shinyo Koeki (Komaki, Japan), respectively. Methanol was obtained from Daejung Chemicals and Metals (Siheung, Korea). All chemicals were used as received without further purifications.

### 2.2. Synthesis of Fe_3_O_4_ and Fe_3_O_4_@ZIF-8 Nanoparticles

Fe_3_O_4_ nanoparticles were synthesized by modifying a previously reported method [27,28]. Typically, 1.35 g of FeCl_3_·6H_2_O was dissolved in 25 mL of ethylene glycol upon stirring for 30 min to form a homogeneous yellow solution. Thereafter, 2.78 g of NaOAc was added and the resulting mixture was stirred for another 30 min. The mixture was transferred into a Teflon-lined stainless-steel autoclave and placed in an oven at 200 °C for 10 h. After cooling to room temperature, the black powder was collected using a magnet, washed with DI water three times, and then stored in methanol prior to use.

For the sonochemical synthesis of the Fe_3_O_4_@ZIF-8 composite, the surface of Fe_3_O_4_ nanoparticles was first modified using an aqueous solution of 0.4 wt.% PSS with the aid of ultrasonic agitation for 30 min. The modified Fe_3_O_4_ (20 mg) was transferred to a glass reactor containing 5 mL of methanol, and then agitated for 2 min. A 13 mm Ti probe of an ultrasonic processor (VCX 130, Sonic and Materials) was placed in the resulting dispersion. Subsequently, ZIF-8 synthesis was carried out under ultrasound irradiation for 30 min with independent supplies of two methanol solutions of 35 mM Zn(NO_3_)_2_ and 280 mM Hmin at a flow rate of 0.5 mL min^−1^ using a syringe pump. The ultrasonic processor was typically operated under 50% amplitude (power) with an irradiation time of 1 s and a relaxation time of 4 s without external cooling. The products were separated using a magnet and washed with MeOH three times. The resultant composites were dried in an oven at 160 °C for 10 h.

For comparison, ZIF-8 [29] and *c*-Fe_3_O_4_@ZIF-8 [22] nanoparticles were prepared using conventional methods without ultrasound irradiation. Briefly, ZIF-8 was synthesized in 30 mL of methanol solution containing 0.312 g of Zn(NO_3_)_2_·6H_2_O and 0.690 g of Hmin at room temperature with a reaction time of 5 h. The resulting white product was separated via centrifugation, washed with MeOH three times, and then dried at 150 °C for 10 h. In the case of *c*-Fe_3_O_4_@ZIF-8, the PSS-modified Fe_3_O_4_ (25 mg) was dispersed in 30 mL MeOH, followed by the addition of 0.149 g of Zn(NO_3_)_2_·6H_2_O and 0.246 g of Hmin. This mixture was used to grow the ZIF-8 crystals at 60 °C for 30 min. *c*-Fe_3_O_4_@ZIF-8 was obtained using the same separation and drying procedure as that used for Fe_3_O_4_@ZIF-8.

### 2.3. Materials Characterization

The microstructures of the samples were observed through scanning electron microscopy (SEM; S-4800, Hitachi, Tokyo, Japan) and transmission electron microscopy (TEM; JEM-3010, Jeol, Tokyo, Japan) [30]. The crystalline structure was determined through powder X-ray diffraction (PXRD; MiniFlex 600, Rigaku, Tokyo, Japan) using an X-ray diffractometer with Cu Kα_1_ radiation (λ = 1.5406 Å). To probe the presence of the chemical functional groups, Fourier transform infrared (FTIR) spectroscopy was performed using a Spectrum Two spectrometer (Perkin–Elmer, Waltham, MA, USA) equipped with an attenuated total reflectance sampling accessory over a wavenumber range of 500–4000 cm^−1^. Nitrogen sorption measurements (Belsorp-mini II, MicrotracBEL, Osaka, Japan) were performed to characterize the specific Brunauer, Emmett, and Teller (BET) surface area. Thermogravimetric analysis (TGA; TGA 4000, Perkin–Elmer) was conducted under an air atmosphere to evaluate the relative content of ZIF-8 in Fe_3_O_4_@ZIF-8. The samples used in the N_2_ sorption and TGA experiments were pretreated in a vacuum oven at 160 °C for 10 h. The magnetism was determined using a vibrating sample magnetometer (MPMS 3, Quantum Design, Darmstadt, Germany) with an external magnetic field ranging from −70 to 70 kOe at 300 K.

### 2.4. Cu(II) Adsorption Study

Batch adsorption experiments were conducted to evaluate the adsorption capacity, removal efficiency, and adsorption kinetics of Fe_3_O_4_@ZIF-8 for Cu^2+^ ions in an aqueous solution. These tests were performed at room temperature and at pH = 5.5. The standard solution of Cu^2+^ with a concentration of 180 mg L^−1^ was prepared by dissolving 0.68 g of Cu(NO_3_)_2_·3H_2_O in deionized water. For each test, 10 mg of Fe_3_O_4_@ZIF-8 was added to 20 mL of the Cu^2+^(aq) solution, followed by short ultrasonic agitation for quick dispersion, and then was shaken at 170 rpm for a specific adsorption time. The Cu concentration was assessed using inductively coupled plasma-optical emission spectrometry (ICP-OES; Avio-200, Perkin–Elmer). The adsorption capacity (*q*) in unit of mg g^−1^ was calculated using the following equation:

*q* = (*C*_0_ − *C*) × *V*/*m* (1)

where *C_0_* and *C* are the initial and final Cu concentrations in mg L^−1^, respectively, *V* is the volume of Cu^2+^(aq) solution in mL, and *m* is the weight of the adsorbent in *g*. The percentage removal efficiency (*R*) was determined using Equation (2):

*R* = 100 × (*C*_0_ − *C*)/*C*_0_ (2)

To study the adsorption kinetics, the *q* values were obtained at different adsorption times in the range of 0–2 h. The removal efficiency was determined from the saturated Cu concentration at an adsorption time of 2 h. The same adsorption tests were carried out to compare the Fe_3_O_4_, ZIF-8, and *c*-Fe_3_O_4_@ZIF-8 samples.

## 3. Results and Discussion

### 3.1. Material Characteristics of Fe_3_O_4_@ZIF-8

**Figure 1:**
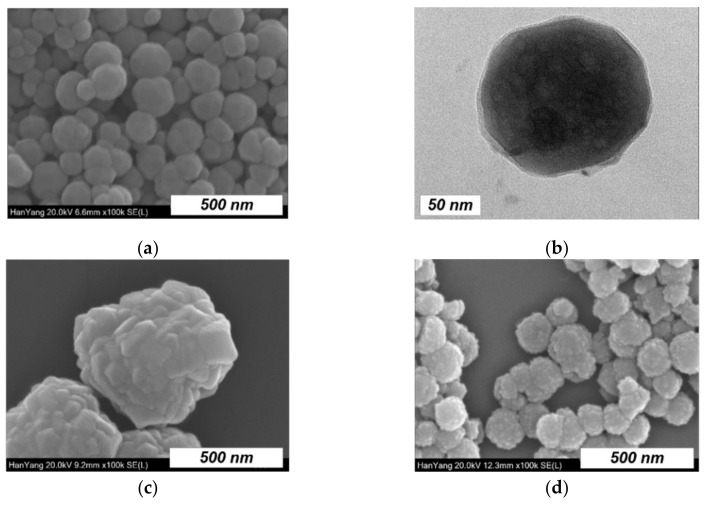
(**a**) Scanning electron microscope (SEM) and (**b**) transmission electron microscopy (TEM) images of Fe_3_O_4_, and SEM images of (**c**) Fe_3_O_4_@ZIF-8 and (**d**) *c*-Fe_3_O_4_@ZIF-8.

Representative SEM and TEM images of the as-synthesized Fe_3_O_4_ are shown in Figure 1a,b, respectively. In general, the Fe_3_O_4_ nanoparticles have a spherical shape with an average diameter of approximately 90 nm. The TEM image reveals voids inside the nanoparticles, which suggests that the Fe_3_O_4_ indeed consists of smaller primary nanocrystals. Figure 1c shows the SEM image of the Fe_3_O_4_@ZIF-8 composite prepared by the sonochemical ZIF-8 synthesis with a reaction time of 30 min. The composite was significantly enlarged up to a size of 675 nm and its surface revealed an uneven, compact morphology that may be formed by in-grown polyhedra of ZIF-8 crystals with a domain size in the range of 50–100 nm. The increase in size and the formation of faceted surfaces imply that Fe_3_O_4_ was well encapsulated by the crystalline ZIF-8 nanoparticles. In contrast, the *c*-Fe_3_O_4_@ZIF-8 nanoparticles prepared via a one-pot solvothermal route (see Figure 1d) display only a slight increase in size compared to the initial Fe_3_O_4_, and the surface morphology is found to be modified by very small nanoparticles (~10 nm). These results indicate that the sonochemical synthesis is more effective than the conventional solvothermal method to produce large Fe_3_O_4_@ZIF-8 nanoparticles with higher ZIF-8 contents.

**Figure 2:**
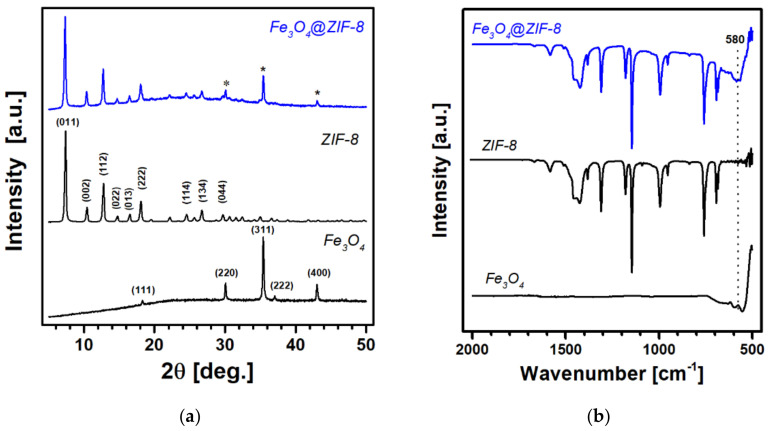
(**a**) Powder X-ray diffraction (PXRD) and (**b**) Fourier transform infrared (FTIR) spectra obtained for Fe_3_O_4_, ZIF-8, and Fe_3_O_4_@ZIF-8. The asterisk symbols indicate three peak positions for Fe_3_O_4_.

The PXRD patterns obtained for the Fe_3_O_4_, ZIF-8, and Fe_3_O_4_@ZIF-8 samples are shown in Figure 2a. The positions of Bragg diffractions by the composite material in 2θ below 30° are in a good agreement with those of ZIF-8 (COD 7111973). The three peaks marked by asterisks above 2θ of 30° can be assigned to the planes of magnetite Fe_3_O_4_ with a cubic inverse spinel structure (JCPDS 19-0629). These results corroborate the formation of highly pure magnetite Fe_3_O_4_ and crystalline ZIF-8 from our synthetic methods described above. In addition, the successful growth of ZIF-8 was also confirmed by FTIR spectroscopy, as shown in Figure 2b. The spectrum for Fe_3_O_4_@ZIF-8 is identical to that of ZIF-8 with the exception for the broad band at 580 cm^−1^ that is attributed to the Fe–O stretching vibrations of the Fe_3_O_4_ nanoparticles [19]. The peaks observed in the region of 600–1700 cm^−1^ are in good agreement with the characteristic vibration bands of ZIF-8 crystals: C = N stretching at 1584 cm^−1^, stretching bands of an imidazole ring at 1350–1500 cm^−1^, and plane bending bands of an imidazole ring at 600–1350 cm^−1^ [17,18].

**Figure 3:**
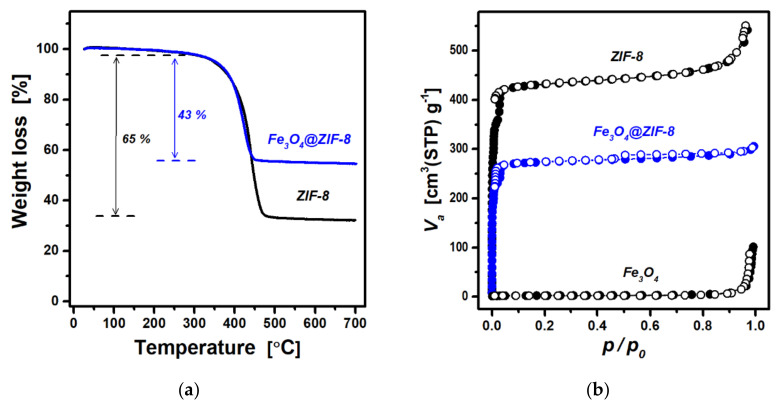
(**a**) Thermogravimetric curves obtained under an air atmosphere and (**b**) nitrogen sorption isotherms (solid circles for adsorption and open circles for desorption).

The relative amount of ZIF-8 in Fe_3_O_4_@ZIF-8 was estimated using TGA under an air atmosphere. Figure 3a shows the thermogravimetric curves obtained for ZIF-8 and Fe_3_O_4_@ZIF-8. These curves exhibit a slight weight loss of 1.5% at <300 °C and a significant weight loss (65% for ZIF-8 and 43% for Fe_3_O_4_@ZIF-8) in the temperature range of 300–500 °C. The former may be ascribed to the evaporation of adsorbed water and remaining solvent molecules, whereas the latter can be interpreted as the pyrolysis of organic ligands of ZIF-8. Assuming that ZIF-8 was completely transformed into ZnO via pyrolysis, the theoretical weight loss of ZIF-8 is 64.2%, which is very close to the observed value (65%). Therefore, the ZIF-8 content in Fe_3_O_4_@ZIF-8 was estimated to be 66 wt.% using the same assumption.

The nitrogen sorption isotherms obtained for ZIF-8, Fe_3_O_4_, and Fe_3_O_4_@ZIF-8 at 77 K are shown in Figure 3b. As expected, the data for pure ZIF-8 display a type I isotherm characteristic of microporous solids. The specific surface area of ZIF-8 obtained using the BET model is 1419 m^2^ g^−1^ and is slightly higher than the previously reported values for ZIF-8 crystals [16,25]. The Fe_3_O_4_ nanoparticles exhibit a very low adsorption up to a high relative pressure (*p/p_0_*~0.9), and the BET surface area is negligible (8 m^2^ g^−1^). Meanwhile, the N_2_ sorption measured for sonochemically prepared Fe_3_O_4_@ZIF-8 displays a very fast uptake at low relative pressures (*p/p_0_*~1 × 10^−4^), and overall shows a similar shape as that for pure ZIF-8. The BET surface area is found to be 896 m^2^ g^−1^. Assuming that the surface area solely depends on the amount of microporous ZIF-8, the relative content of the ZIF-8 in Fe_3_O_4_@ZIF-8 composite is estimated to be 63 wt.%. Note that this value is reasonably close to the ZIF-8 content of 66 wt.% determined by TGA. On the other hand, the Fe_3_O_4_@ZIF-8 isotherm shows a perceptive hysteresis loop in the region of *p/p_0_* = 0.45 − 0.9, suggesting the presence of mesopores. The high surface area and dual micro/mesoporosity in Fe_3_O_4_@ZIF-8 are desirable properties of an adsorbent for high adsorption capacity and fast mass transport.

**Figure 4:**
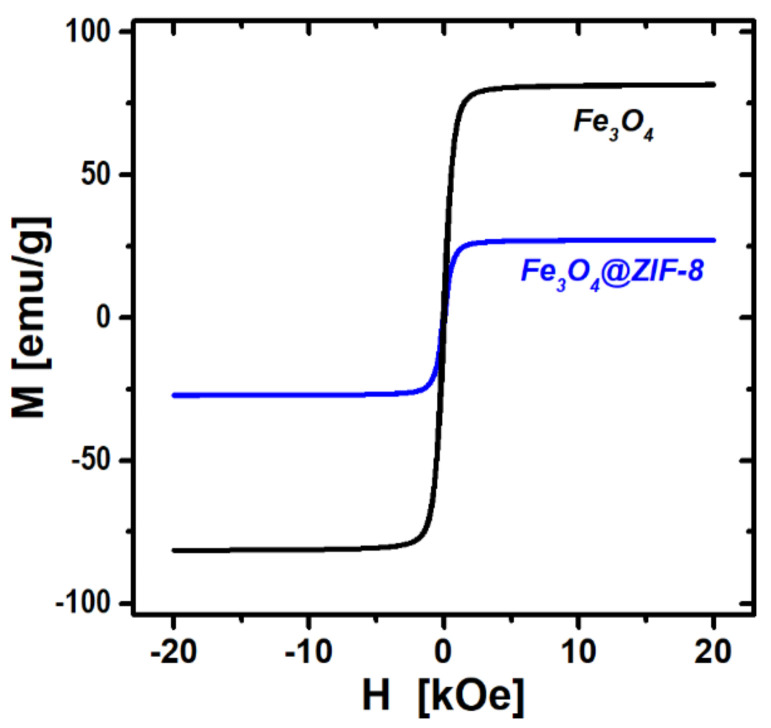
Magnetization curves obtained for Fe_3_O_4_ and Fe_3_O_4_@ZIF-8.

Figure 4 shows the magnetic hysteresis loops measured for Fe_3_O_4_ and Fe_3_O_4_@ZIF-8, which displays superparamagnetic characteristics with negligible remanent magnetization at zero external magnetic field. The saturation magnetization of Fe_3_O_4_ is 82 emu g^−1^. After the ZIF-8 encapsulation, the magnetic response of Fe_3_O_4_@ZIF-8 decreases to 27 emu g^−1^ due to the addition of non-magnetic ZIF-8. When the Fe_3_O_4_ content of 32.5 wt.% was taken into consideration, the Fe_3_O_4_-specific saturation magnetization is almost identical to that of pure Fe_3_O_4_, indicating that the magnetization has not been affected by the ZIF-8 modification step. The strong magnetic response would allow the Fe_3_O_4_@ZIF-8 particles to be readily separated from an aqueous solution using a magnet within a very short time (<1 min). A redispersion of the composite particles after adsorptive removal is also possible by mild agitation in the absence of a magnetic field.

### 3.2. Effects of the Reaction Time and Sonication Power on the Formation of Fe_3_O_4_@ZIF-8

**Figure 5:**
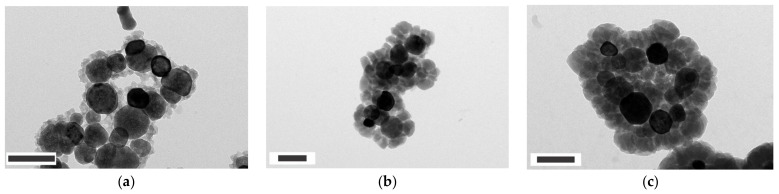
TEM images of Fe_3_O_4_@ZIF-8 observed at different reaction times: (**a**) 10, (**b**) 20, and (**c**) 30 min. Scale bars represent 200 nm.

**Figure 6:**
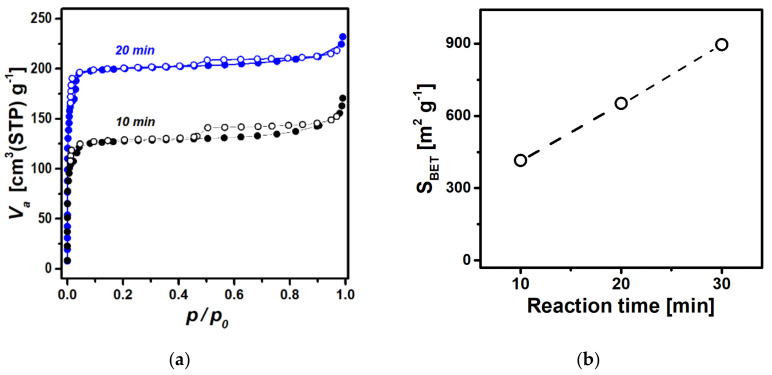
(**a**) Nitrogen sorption isotherms obtained for Fe_3_O_4_@ZIF-8 at different reaction times of 10 and 20 min. (**b**) Plot of BET surface area vs. reaction time.

**Figure 7:**
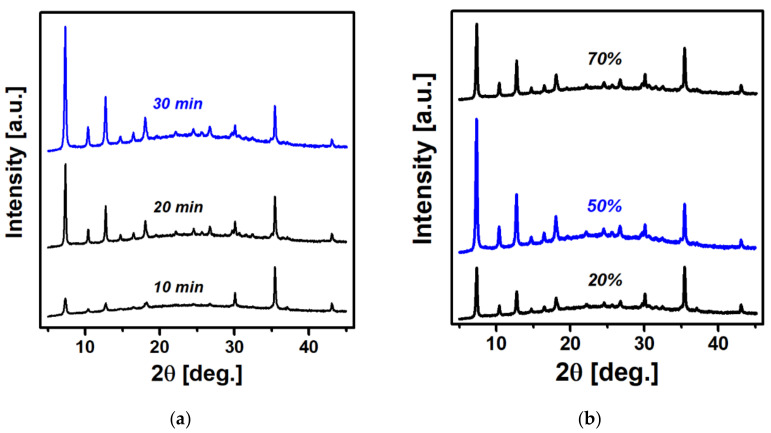
PXRD patterns obtained for Fe_3_O_4_@ZIF-8 synthesized at (**a**) different reaction times of 10, 20, and 30 min and (**b**) different sonication powers of 20%, 50%, and 70%.

To gain an insight into the sonochemical formation of ZIF-8 on Fe_3_O_4_, the microstructures were examined using TEM for Fe_3_O_4_@ZIF-8 obtained at different reaction time. Figure 5a–c shows the TEM images of the Fe_3_O_4_@ZIF-8 samples prepared by the reaction times of 10, 20, and 30 min, respectively. The composites clearly exhibit core-shell structures, i.e., the encapsulation of Fe_3_O_4_ nanoparticles (black) by a ZIF-8 layer (gray). As the reaction time increases, the shell thickness increases from 30 nm at 10 min to 80 nm at 20 min to 110 nm at 30 min. Furthermore, it is evident from the images that voids formed by interlinked magnetic nanoparticles are filled by growing ZIF-8 shells as the reaction time increases. The variation in the microstructures as a function of reaction time was also investigated using N_2_ sorption experiments. Figure 6a,b shows the sorption isotherms and changes in BET surface area, respectively, for the samples prepared with different reaction times. The surface area linearly increases as a function of the reaction time, indicating the continued growth of ZIF-8 in the Fe_3_O_4_@ZIF-8 system. Moreover, the hysteresis loop in N_2_ isotherms becomes less pronounced as the reaction time increases. Again, this can be interpreted as ZIF-8 shells growing and filling up the interparticle mesopores as the ultrasonic reaction time increases. This growth behavior was further verified by fitting the observed PXRD data with patterns calculated from ZIF-8 and Fe_3_O_4_. As shown in Figure 7a, the relative amount of ZIF-8 in the composite increases from 23% at 10 min to 47% and 67% at 20 and 30 min, respectively. The material characterization data above collectively suggest and prove that sonochemical treatments result not only in the synthesis of ZIF-8 crystals on Fe_3_O_4_ nanoparticles, but also in a progressive growth of the ZIF-8 shells that gradually fuse to fill up the interparticle voids. Thus, the sonochemical route can provide an opportunity to control the shell thickness and pore dimensions in core–shell Fe_3_O_4_@ZIF-8 by means of the reaction time.

ZIF-8 formation on Fe_3_O_4_ is also strongly affected by the sonication power used in the sonochemical synthesis. Figure 7b shows the PXRD patterns obtained for the Fe_3_O_4_@ZIF-8 products prepared over 30 min at sonication powers of 20%, 50%, and 70%. A quantitative analysis was carried out for these data using the calculated patterns of ZIF-8 and Fe_3_O_4_. According to the results, the ZIF-8 content increases from 39 to 52 wt.% on going from 20% to 50% power but decreases to 47 wt.% at 70% sonication power. This analysis indicates that the maximum loading of ZIF-8 on Fe_3_O_4_ is not achieved at the highest available power of the sonication. The formation of ZIF-8 itself from the mixture of precursors should be enhanced as the power increases. However, the breakdown of Fe_3_O_4_@ZIF-8 could also occur as the available energy delivered by the ultrasound waves exceeds a certain level. In fact, a milky appearance was observed in the supernatant solution after the separation of the composites synthesized at 70% power, which suggests the detachment and/or homogeneous growth of small ZIF-8 nanoparticles. These results demonstrate that the sonication power is an important synthesis parameter that should be controlled to obtain a magnet-responsive Fe_3_O_4_@ZIF-8 composites with a high ZIF-8 content. Consequently, a mass production of our magnetic Fe_3_O_4_@ZIF-8 absorbent will be readily possible through the growth of ZIF-8 nanocrystals on magnetic Fe_3_O_4_ under the optimized sonochemical conditions.

### 3.3. Adsorption of Cu(II) Ions by Fe_3_O_4_@ZIF-8

**Figure 8:**
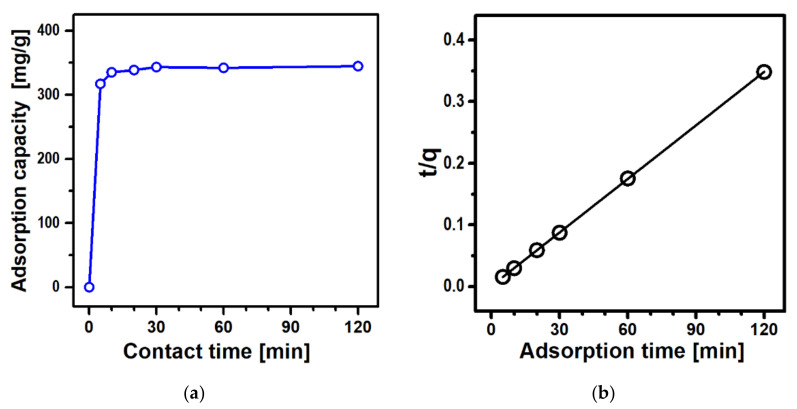
(**a**) Effect of contact time and (**b**) pseudo-second-order kinetic model for Cu^2+^ adsorption by Fe_3_O_4_@ZIF-8.

Adsorption experiments utilizing Fe_3_O_4_@ZIF-8 were carried out using an aqueous solution of Cu^2+^ with a concentration of 180 mg L^−1^. Figure 8a shows the Cu^2+^ adsorption capacity as a function of the contact time. The adsorption takes place quickly for the first 10 min and then reaches a saturated state within 30 min. The uptake capacity at 10 min is 335 mg g^−1^ (97% of the saturation level), which implies that the contact time of 10 min is enough to adsorb most of the Cu^2+^ ions present in water. The very fast adsorption is comparable to that of pure ZIF-8 [16], which can be attributed partly to the dual micro/mesoporosity of Fe_3_O_4_@ZIF-8 and partly to the chemical interactions between the Cu^2+^ adsorbate and amine moieties on the surface of the ZIF-8 shell. When compared to that of other representative magnetic Cu^2+^ adsorbents (see Table 1), the adsorption time of Fe_3_O_4_@ZIF-8 is extremely short. Moreover, summarized in Table 1 are the maximum adsorption capacities of the magnet-responsive adsorbents. The adsorption uptake of our Fe_3_O_4_@ZIF-8 exceeds others by a large margin. As suggested above, the high uptake capacity is attributed to the high surface area of our Fe_3_O_4_@ZIF-8 adsorbent which again is a result of high ZIF-8 content in the composite.

The time-dependent adsorption was further analyzed using pseudo-first-order and pseudo-second-order kinetic models [37]. The obtained data are in good agreement with the pseudo-second-order equation shown below:

(3) $$\frac{t}{\;q\;}=\frac{1}{\;k_{2}q_{e\;}^{2}}+\frac{t}{\;q_{e}}$$

where *q* is the adsorption capacity, *t* is the contact time, *q_e_* is the adsorption capacity at equilibrium, and *k_2_* is the equilibrium rate constant. The correlation coefficient obtained using linear regression analysis is R^2^ = 0.99999, displaying nearly perfect linearity, as shown in Figure 8b. The equilibrium adsorption capacity obtained by this method is *q_e_* = 346 mg g^−1^ which agrees very well with the saturated value of 345 mg g^−1^ obtained after a contact time of 2 h. The pseudo-second-order kinetics suggests that the rate-limiting step is chemisorption [37]. The adsorption mechanism can be interpreted with both the ion exchange reactions of Cu^2+^ ions with Zn^2+^ in ZIF-8 and the coordination reactions between Cu^2+^ and the nitrogen atom in 2-methylimidazole, as proposed previously [16].

**Figure 9:**
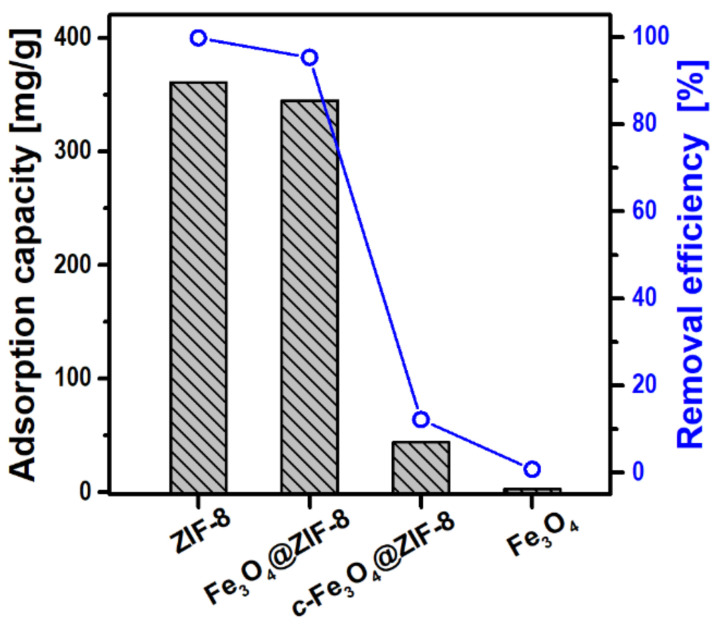
Adsorption capacity and removal efficiency of Fe_3_O_4_@ZIF-8, *c*-Fe_3_O_4_@ZIF-8, ZIF-8, and Fe_3_O_4_.

Figure 9 shows the magnitudes of the adsorption capacity and removal efficiency for the Fe_3_O_4_@ZIF-8 adsorbent along with ZIF-8, Fe_3_O_4_, and *c*-Fe_3_O_4_@ZIF-8 samples for comparison. Fe_3_O_4_@ZIF-8 exhibits an adsorption capacity of 345 mg g^−1^ and removal efficiency of 95.3%, which are only slightly lower than those of pure ZIF-8 (361 mg g^−1^ and 99.7%), but significantly higher than those of *c*-Fe_3_O_4_@ZIF-8 or Fe_3_O_4_. The great difference between Fe_3_O_4_@ZIF-8 and *c*-Fe_3_O_4_@ZIF-8 samples is understandable with the variation in ZIF-8 content. Because the Fe_3_O_4_ component in the composite is inactive toward Cu^2+^ adsorption, the value of our Fe_3_O_4_@ZIF-8 comparable to that of pure ZIF-8 is highly surprising. These results should be seen as synergetic effects of the high ZIF-8 loading and the presence of micro/mesoporosity. A similar case was reported in a hierarchically structured ZIF-8 system displaying very high uptake capacity for the toxic arsenate in water [38].

## 4. Conclusions

A new sonochemical strategy was proposed to synthesize magnetic particle-MOF composites. This approach resulted in the rapid and efficient production of high-quality Fe_3_O_4_@ZIF-8 with a high ZIF-8 content, while minimizing the necessity for chemicals. The sonochemical method is also advantageous to create well-dispersed and uniform Fe_3_O_4_@ZIF-8 particles because the magnetic Fe_3_O_4_ nanoparticles are subject to aggregation under no agitation. The synthetic parameters, namely, reaction time and sonication power, can be readily controlled to obtain the composite material with different ZIF-8 loadings (shell thickness) and distinct micro/mesoporosity. The superparamagnetic Fe_3_O_4_@ZIF-8 with a high ZIF-8 loading was proved to be an excellent adsorbent for the removal of toxic copper ions from water. The functional nanomaterial may also be utilized in other applications, such as drug delivery, sensing, bioimaging, and heterogeneous catalysis.

## Figures and Tables

**Table 1:** Adsorption performances of representative magnet-responsive Cu^2+^ adsorbents.

Adsorbents ^1^	Synthesis Method	Adsorption Time (min)	Maximum Adsorption Capacity (mg g^−1^)	Reference
rGO-PDTC/Fe_3_O_4_	coprecipitation	45	114	[31]
MCGO	coprecipitation	-	217	[32]
XMCS	grafting	-	34.5	[33]
Fe_3_O_4_@GO/MnO_x_	hydrothermal	300	62.6	[34]
Fe_3_O_4_@ZIF-8	wet synthesis	120	46.8	[35]
Fe_3_O_4_@zeolite NaA	coprecipitation	24	86.5	[36]
Fe_3_O_4_@ZIF-8	sonochemistry	10	335	this work

^1^ rGO-PDTC: dithiocarbamate-modified reduced graphene oxide; MCGO: magnetic chitosan/graphene oxide; XMCS: xanthate-modified magnetic chitosan.

## Data Availability

The data presented in this study are available on request from the corresponding authors.
